# Strategies of Macrophages to Maintain Bone Homeostasis and Promote Bone Repair: A Narrative Review

**DOI:** 10.3390/jfb14010018

**Published:** 2022-12-29

**Authors:** Yingkun Hu, Jinghuan Huang, Chunying Chen, Yi Wang, Zhuowen Hao, Tianhong Chen, Junwu Wang, Jingfeng Li

**Affiliations:** 1 Department of Orthopedics, Zhongnan Hospital of Wuhan University, Wuhan 430000, China; 2 Department of Orthopedic Surgery, Shanghai Jiao Tong University Affiliated Sixth People’s Hospital, Shanghai 200000, China

**Keywords:** bone homeostasis, bone remodeling, macrophage, macrophage polarization, bone repair

## Abstract

Bone homeostasis (a healthy bone mass) is regulated by maintaining a delicate balance between bone resorption and bone formation. The regulation of physiological bone remodeling by a complex system that involves multiple cells in the skeleton is closely related to bone homeostasis. Loss of bone mass or repair of bone is always accompanied by changes in bone homeostasis. However, due to the complexity of bone homeostasis, we are currently unable to identify all the mechanisms that affect bone homeostasis. To date, bone macrophages have been considered a third cellular component in addition to osteogenic spectrum cells and osteoclasts. As confirmed by co-culture models or in vivo experiments, polarized or unpolarized macrophages interact with multiple components within the bone to ensure bone homeostasis. Different macrophage phenotypes are prone to resorption and formation of bone differently. This review comprehensively summarizes the mechanisms by which macrophages regulate bone homeostasis and concludes that macrophages can control bone homeostasis from osteoclasts, mesenchymal cells, osteoblasts, osteocytes, and the blood/vasculature system. The elaboration of these mechanisms in this narrative review facilitates the development of macrophage-based strategies for the treatment of bone metabolic diseases and bone defects.

## 1. Introduction

As one of the most critical tissues in the human body, bone protects internal organs, provides mechanical support and muscle attachment, and is a storehouse of calcium and phosphorus. Even after skeletal maturation, the replacement of new bone with old bone occurs periodically. In bone remodeling, fatigued or impaired bone is removed by osteoclasts, followed by new bone formation by osteoblasts. Over the course of a person’s lifetime, this process will continue. The lifelong process occurs asynchronously through temporary equilibrium anatomic structures called basic multicellular units (BMUs), which are composed of different cell types such as osteoblasts, osteoclasts, and osteocytes [1,2]. BMUs play a crucial role in maintaining bone quality and structural integrity and in regenerating it physiologically.

Bone is a composite or hybrid material composed of organic (primary collagen type I) and inorganic (main hydroxyapatite). Collagen type I, the predominant organic component of the native bone matrix, is the typical triple helix collagen consisting of two α1 chains and one α2 chain, whose deposition could be conspicuously important in boosting new bone growth in vivo and facilitating de novo mineralization [3,4,5]. In the organic matrix, collagen type I accounts for roughly 95% of the proteins, with the remainder made up of other proteins and proteoglycans, such as osteonectin, osteopontin, and osteocalcin [6]. As a calcium phosphate mineral, hydroxyapatite (HA) is the principal inorganic component of human bones, and their c-axis lines up regularly along collagen fibers [7]. The complementary functions of osteoblasts, which promote ossification; osteoclasts, which cause bone destruction; and osteocytes, which regulate bone metabolism and sense mechanical forces, work together to maintain the homeostasis of bone remodeling [8]. There are several successive phases involved in bone remodeling: detection of microdamage to bone by osteocytes, provision of energy for bone resorption by osteoclasts, differentiation of mesenchymal stem cells (MSC) into osteoblasts, regeneration of bone by osteoblasts, and the completion of bone formation [9]. In adult humans, an estimated 10% of the existing skeleton is replaced annually in the normal skeletal microenvironment [10]. In the long-term inflammatory microenvironment caused by aging in the elderly, bone resorption exceeds bone formation, causing bone loss. A significant risk factor for bone loss is chronic stress. There is no doubt that macrophage polarization is correlated with inflammatory microenvironments [11,12,13]. Recently, it has also been proposed that macrophages are a third cellular component of bone in addition to osteogenic spectrum cells and osteoclasts [14]. Thus, the regulation of bone homeostasis may be carried out by macrophages.

**Figure 1:**
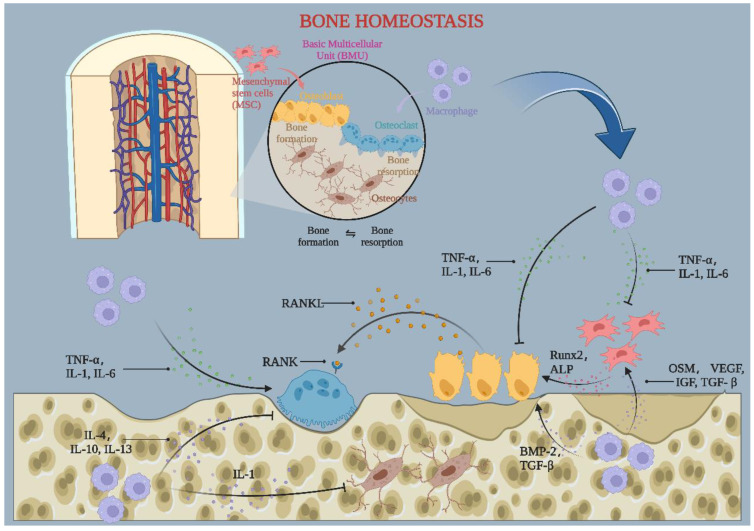
The role of macrophages in bone homeostasis. Macrophages fuse and differentiate into osteoblasts. Macrophages secrete cytokines, including TNF-α, IL-1, and IL-6, which inhibit bone formation and promote bone resorption. They can also release cytokines such as IL-4, IL-10, and IL-13, inhibiting osteoclast differentiation. OSM, VEGF, IGF, TGF-β, and BMP-2 are secreted to stimulate osteoblast differentiation. By BioRender (AZ24OOGN51).

Macrophages are present in almost all tissues as innate immune cells and are essential to maintaining normal tissue homeostasis. Macrophages promote tissue homeostasis during oxidative stress by phagocytosing invading microorganisms, increasing inflammatory responses, and recruiting additional immune cells. There are two leading polarization states of macrophages: M1 macrophages are polarized based on their classical activation, whereas M2 macrophages are alternatively activated. In recent years, much attention has been paid to macrophages owing to their involvement in immunity, tissue repair, and tissue homeostasis. Furthermore, their key role in normal bone homeostasis is receiving increasing attention. The current study found that macrophages of different phenotypes interact with osteoclasts and osteoblasts at various levels to regulate normal bone homeostasis (Figure 1). However, abnormal polarization of macrophages induced by oxidative stress stimulation can lead to an imbalance in bone homeostasis and bone loss. As research has progressed, many discoveries have been made about the mechanisms by which macrophages control bone homeostasis. Understanding these findings will help us better understand bone metabolic disorders and suggest new treatment options.

The complexity of the signaling pathways involved in bone homeostasis makes it difficult for us to quantify them using a uniform standard. Therefore, we have provided an overview of macrophage-mediated bone homeostasis using a narrative review format. Database searches were performed using the database Medline with macrophages and bone as keywords. The literature was selected from the last five years and irrelevant material was manually excluded. In this narrative review, we first describe the functions of various cells involved in the regulation of bone homeostasis, as well as transcriptional regulators and markers. Then we illustrate the regulation of bone homeostasis by macrophages through the macrophage–osteoclast axis, macrophage–MSCs axis, macrophage–osteoblast axis, macrophage–osteocyte axis, and macrophage–blood/vasculature system axis. Finally, we provide an outlook on therapies using macrophages for the treatment of bone metabolic diseases and bone defect repair.

This manuscript aims to help elucidate the mechanisms by which macrophages regulate bone homeostasis. This has the potential to help solve two fundamental orthopaedic challenges: bone loss and bone defects. The development of drugs targeting macrophage-associated cytokines could be a key therapy in reducing bone loss. Moreover, the use of macrophage strategies on applied biomaterials also contributes to bone regeneration and the healing of bone defects. In conclusion, macrophage strategies are likely to be key in the treatment of bone loss and bone defects.

## 2. Physiological Bone Remodeling: Involvement of Multiple Cell Types

**Figure 2:**
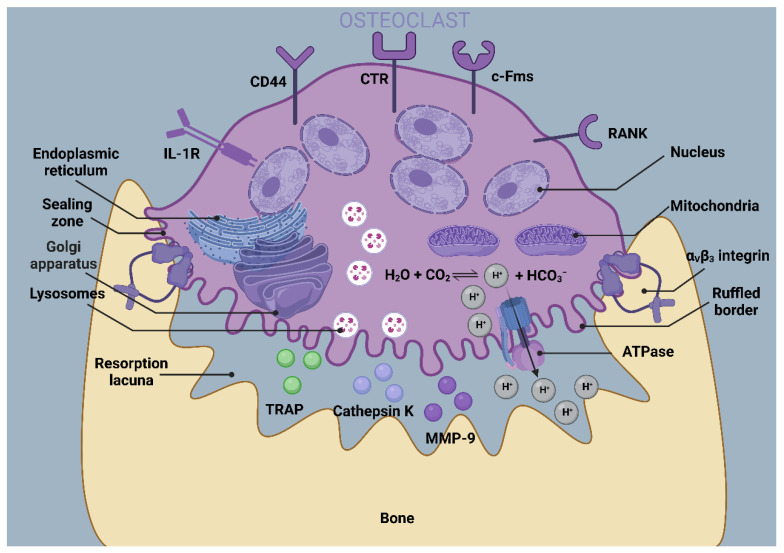
Bone resorption activity of osteoclasts. Osteoclasts resorb bone matrix by releasing H^+^, Cl^−^, cathepsin K, and MMPs in the region of bone resorption, in which there are numerous mitochondria. Inorganic mineralized constituents of the bone matrix are absorbed in the acidic microenvironment, while organic compounds are exposed to protease-catalyzed degradation. The αVβ3 integrin can promote the formation of actin rings. The area of the resorption surface is closed by an actin ring to prevent leakage of acid and catabolic enzymes, thus enabling precise control of the bone transition site. At the same time, the cell membrane of osteoclasts in the area of bone resorption folds up to form the ruffled border to improve the efficiency of bone resorption. By BioRender (HO24OOGK2P).

**Figure 3:**
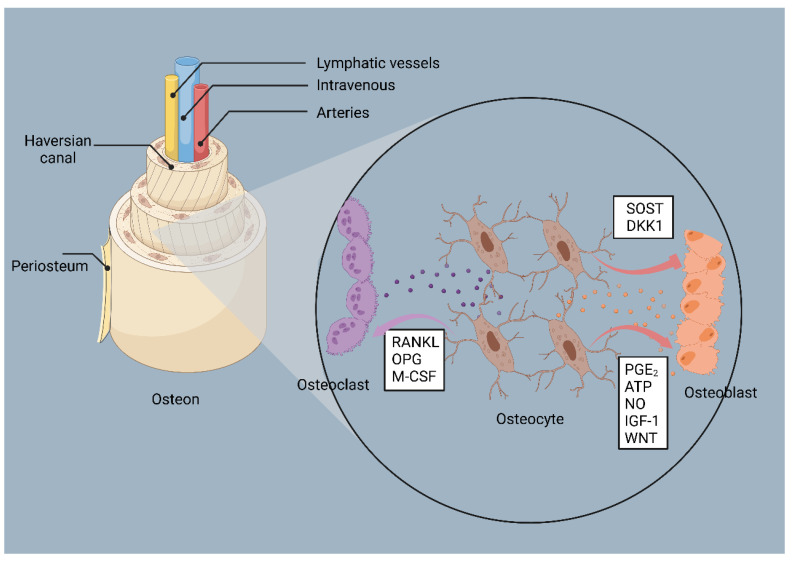
Osteocytes coordinate the activities of osteoblasts and osteoclasts. Osteocytes control osteoclast production by adjusting the release of RANKL, OPG, and M-CSF. Osteocytes produce PGE_2_, ATP, NO, IGF-1, and WNT to promote osteoblast formation and inhibit osteoblast differentiation via SOST and DKK1. By BioRender (OC24OOGPJD).

**Figure 4:**
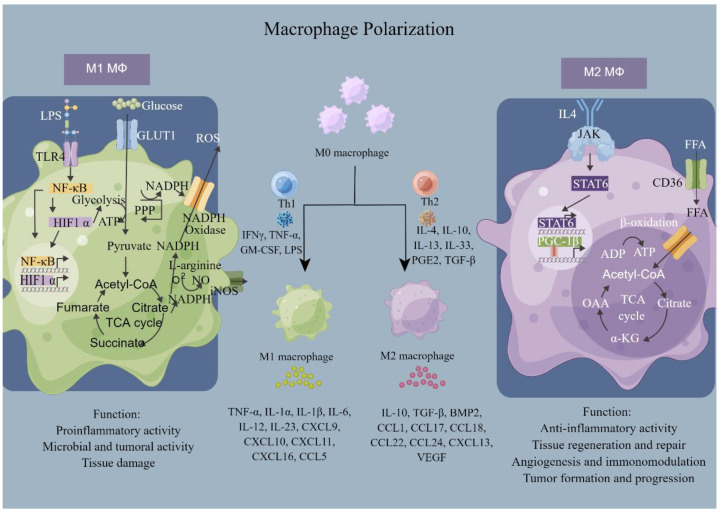
Macrophage polarization, as well as metabolic reprogramming. Macrophages are polarized to M1 or M2 after exposure to different stimulating factors. Macrophages with different polarizations produce various cytokines and perform multiple functions in tissue homeostasis. By Figdraw (REG8221117054600062688).

Bone homeostasis is controlled by a variety of cells, including osteoclasts, bone marrow mesenchymal stem cells (BMSCs), osteoblasts, osteocytes, and macrophages. Their origin, functions, and key signals are summarized in Table 1. In them, osteoclasts are responsible for the resorption of the bone matrix (Figure 2). BMSCs are a class of cells present in the bone marrow that have multidirectional differentiation potential and are capable of contributing to osteoblastogenesis. Osteoblasts are responsible for bone formation during bone homeostasis and they mature through four sequential processes: lineage orientation, cell proliferation marked by histone expression, matrix maturation with the presentation of extracellular matrix-associated proteins, and mineralization induced by osteocalcin [15]. Mature osteoblasts produce collagen type I, bone morphogenetic protein (BMP), osteocalcin, osteonectin, proteoglycans, and alkaline phosphatase (ALP). Osteoblasts produce osteoid by releasing collagen, which gradually mineralizes and, eventually, osteoblasts either suffer apoptosis or transdifferentiate to osteocytes or bone-lining cells [16]. Osteocyte protrusions connect and communicate with each other as conduits, forming an independent “traffic system” to deliver oxygen, nutrients, and signaling molecules (Figure 3). Upon sensing various environmental stimuli, macrophages can be polarized into a classically activated or pro-inflammatory state (M1) and an alternative activated or anti-inflammatory state (M2) (Figure 4). However, current studies have revealed that the subclasses of macrophages that can be triggered are not only M1 and M2, but also include several phenotypes, such as M2a, M2b, M2c, and M2d [17]. Macrophages can maintain bone homeostasis by secreting cytokines, sending paracrine signals, or interacting directly with other cells in the bone.

## 3. Transcriptional Regulation and Markers in Bone Homeostasis

### 3.1. Transcriptional Regulation of Osteoclasts

**Figure 5:**
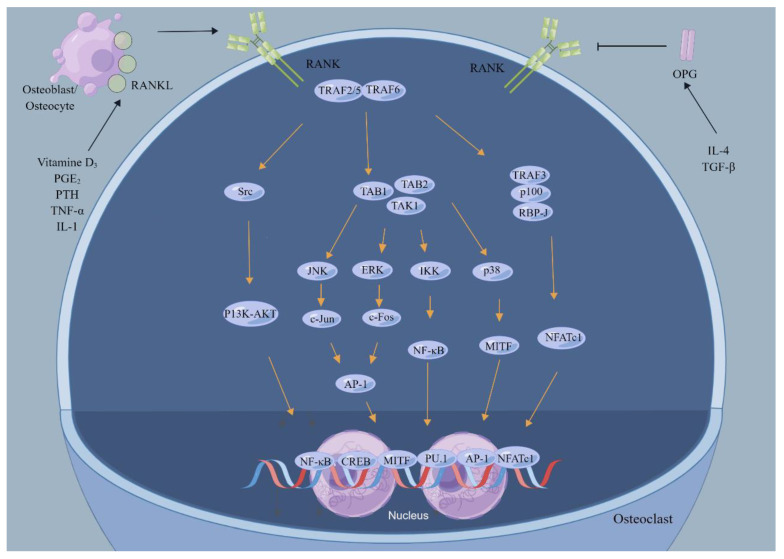
The RANKL/RANK signaling pathway in osteoclasts. RANKL produced by osteoblasts or osteocytes binds to RANK on osteoclasts and activates the RANKL/RANK signaling pathway. Subsequently, it mediates the expression of a series of genes within osteoblasts and promotes osteoclast differentiation. By Figdraw (REG8221022063500114351).

Activation of osteoclasts is directly regulated by nuclear factor κB ligand (RANKL) (Figure 5). RANKL, also called osteoclast differentiation factor (ODF), TNF-related activation-induced cytokine (TRANCE), osteoprotegerin ligand (OPGL), and TNF ligand superfamily member 11 (TNFSF11), is another essential cytokine regulating the maturation of osteoclasts, which has a vital role for bone remodeling [40]. RANKL, a homotrimeric transmembrane protein that is a critical member of the TNF superfamily, is an osteoclast differentiation factor expressed by osteoblasts on their cell membranes, which can be cleaved matrix metalloproteinases (MMPs) to present a soluble form [21]. Numerous growth factors, hormones, and cytokines can adjust RANKL expressions through the canonical regulatory mechanism in osteoblasts, such as parathyroid hormone, vitamin D3, estrogen, and inflammatory cytokines. M-CSF-dependent osteoclast precursor cells are differentiated into mature osteoclasts in the company of RANKL, which binds to the specific receptor RANK via cell–cell interaction. All of the RANK, a type I homotrimeric transmembrane protein with a high homology with CD40, which belongs to the TNF receptor superfamily, contain four cysteine-rich domains (CRDs) [41]. The RANKL signaling pathway is summarized in [42,43,44]. Like M-CSF, RANKL participates in the differentiation of osteoclast precursor cells and bone matrix resorption through MAPKs, including extracellular signal-regulated kinase (EPK), c-Jun amino-terminal kinase (JNK), and p38 signaling pathways.

Interestingly, some recent studies have demonstrated that M-CSF- and RANKL-induced MAPK signaling pathways differ in extent and duration. M-CSF has a more marked and persistent induction of ERK phosphorylation, and M-CSF is more favorable to phosphorylate JNK, while RANKL induces more sustained p38 phosphorylation [45]. M-CSF and RANKL act differently on osteoclast precursor cells due to these differences in activating MAPKs: MAPK signalings triggered by M-CSF are primarily associated with the proliferation and survival of osteoclast precursor cells, while MAPK signalings activated by RANKL mainly contribute to osteoclast differentiation. RANKL-induced expression of the β3 integrin subunit is also required for αVβ3-mediated binding to the bone matrix. Most of these signaling pathways are thought to affect osteoclasts through a master transcription factor, NFATc1, which drives the expression of many osteoclast genes. The secretion of osteoclast protein hydrolase, anti-tartaric acid phosphatase, and collagenase histone K are all dependent on NFATc1-mediated RANKL signaling. Blockage of the RANKL signaling pathway impedes osteoclast formation, making the RANKL signaling pathway a potent target for treating osteoporotic bone loss and other skeletal metabolic diseases. A unique study has recently shown that vesicular RANK secreted by mature osteoclasts binds to RANKL in osteoblasts and enhances bone formation by activating RANKL reverse signaling, which triggers the mTOR pathway and Runx2 [46]. This pioneering work shows that the RANKL signaling pathway is a two-way signaling regulatory system that may have a more critical role in regulating bone metabolism. RANKL is not only closely related to bone homeostasis, but also has been found to be upregulated in bone metabolic diseases, such as arthritis and prosthesis loosening, which may be associated with the development of these diseases. Denosumab, a fully humanized monoclonal antibody to RANKL, is demonstrating a good application prospect in the treatment of osteoporosis [47].

Osteoprotegerin (OPG), also called osteoclastogenesis inhibitory factor (OCIF) and TNF receptor superfamily member 11B (TNFRSF11B), is a secreted glycoprotein usually produced by B lymphocytes and OBs in the bone [48]. OPG works as a decoy receptor negatively regulating RANK/RANKL-induced osteoclast proliferation and differentiation by binding to RANKL. A higher binding capacity exists between OPG and RANKL than between RANK and RANKL, preventing RANKL from binding to RANK, so the ratio of OPG/RANK determines the differentiation of osteoclasts [49]. Several hormones, cytokines, and growth factors can affect the expression of OPG in osteoblasts, such as estrogen, 1,25(OH)_2_ vitamin D_3_, and TNF [30]. Osteocytes and osteoblasts signaled by PTH receptors can increase RANKL/OPG ratios, which may be an essential mechanism for bone resorption [50]. Several newer studies have suggested that local production of OPG is more critical for bone homeostasis than circulating OPG [51].

### 3.2. Transcriptional Regulation of Osteoblasts

All osteoblast proliferation and differentiation stages require Runx2 and OSX transcription factors, which activate genes in bone matrix proteins, such as collagen type I, osteocalcin, and osteopontin to facilitate skeletal formation [35,52]. Many studies have demonstrated the critical role of Runx2 and OSX in osteogenic differentiation. Runx2 is at the crossroads of most regulating systems controlling osteoblast differentiation, such as BMP, WNT, Notch, Hedgehog, and FGF [53]. There is some evidence that Runx2 regulates OSX (SP7) upstream, and Runx2 can reinforce OSX expression [50]. In addition to Runx2, signaling pathways containing BMP and IGF can also regulate the expression of Osterix, while Osterix can also regulate its own expression through feedback regulatory mechanisms [54]. Although multitudinous experiments have been carried out to learn Runx2 and OSX, there are still many mechanisms that are not fully understood, such as whether there are differences in the functions of the different isoforms of these two transcription factors. A large number of signaling factors control Runx2 and OSX transcription in osteoblasts, thus regulating differentiation and survival.

BMSCs’ differentiation into osteoblasts is promoted by Runx2, one of the most crucial osteogenic transcription factors. It is derived from the Runt domain gene family, also known as core binding factor α1 (Cbfa1), polyomavirus enhancer binding protein 2 (PEBP2αA), and acute myeloid leukemia factor 3 (AML3). The Runt domain gene family includes three members: the Runx1 gene, the Runx2 gene, and the Runx3 gene. The Runx1 gene is associated with hematopoiesis and angiogenesis. The Runx3 gene plays an essential role in neurogenesis and tumor suppression. The Runx2 gene is not only closely related to the metastasis and invasion of breast cancer, prostate cancer, thyroid cancer, colon cancer, lung cancer, and other tumor cells, but also plays a vital role in the differentiation of osteoblasts in the process of bone metabolism [55].

Bone formation and differentiation of mesenchymal cells into pre-osteoblasts are dependent on Runx2. Runx2 is regulated by 1,25(OH)_2_ vitamin D_3_ (ViD_3_), which induces the expression of Runx2 in human osteoblasts [56]. Mice with a deletion in the C terminus of the Runx2 gene die at the end of their embryos and exhibit a loss of intramembranous and intrachondral osteogenesis [31]. Runx2-deficient mouse skull cells express a shallow level of immature osteoblast markers (alkaline phosphatase and osteonectin) and do not express mature osteoblast markers (osteopontin and osteocalcin) [57]. In contrast, overexpression of Runx2 in mice increases the secretion of alkaline phosphatase, osteocalcin, and osteopontin. However, at the same time, it will also lead to a large number of immature osteoblasts because the differentiation of osteoblasts remains at the primary level, and further differentiation is hindered [58]. Therefore, the expression of Runx2 needs to decrease in the osteogenic differentiation process. The expression of Runx2 is weak in undifferentiated mesenchymal cells, increased in preosteoblasts, and has the highest level in immature osteoblasts. During the maturation of osteoblasts, Runx2 expression gradually decreases [59]. It is essential that Runx2 levels decrease in osteoblasts in order for them to mature and keep their function. Besides, Runx2 can also induce OPG binding to RANKL, thus reducing the production of osteoclasts.

Osterix, also known as SP7, is a transcription factor of the zinc finger structure specifically expressed by osteoblasts and belongs to the family of specific proteins. Osx plays an important role in the directional differentiation of pre-osteoblasts into immature osteoblasts, which is essential for activating osteogenesis-specific genes that support bone formation. Osteopontin, osteocalcin, and other osteoblast markers are not expressed or are expressed in low amounts in mouse embryos with Osx gene deletion. In addition, mature osteoblasts are entirely absent, and normal bone is not formed in the embryo. Runx2 is generally expressed in Osterix-deficient mice, but Osterix is not significantly expressed in mouse embryos with a Runx2 gene deletion. It is suggested that Runx2 may be an upstream regulator of Osterix, and the transcription of Osterix is positively regulated by Runx2 [60]. Runx2 may activate the Osterix promoter by binding to Runx2 binding elements near the Osterix promoter. In addition to Runx2, signaling pathways, such as BMP and insulin-like growth factor (IGF1), can also regulate Osterix expression [61]. This may be one of the mechanisms by which these molecules promote osteogenic differentiation.

### 3.3. Markers of Bone Formation

#### 3.3.1. Osteocalcin (OCN)

OCN is usually considered a marker of late bone formation. Osteoblasts secrete OCN expressly and incorporate it into the extracellular matrix, making it the most abundant non-collagenous protein in bone [62]. However, osteocalcin expression was highest in less mature osteoblasts close to the bone-forming surface. When bone formation increases, osteoblast production of OCN also increases. During this process, a small fraction of OCN is released into the blood; therefore, the concentration of OCN in the blood is thought to correlate with the rate of bone formation. OCN is essential for the alignment of Bap parallel to collagen fibers, which maintains longitudinal bone strength in long bones by reorienting Bap parallel to collagen fibers, and the c-axis orientation of Bap crystals in OCN-deficient mice is severely disrupted. It is believed that OCN inhibits bone formation, although its function has not been fully elucidated. Karsenty and his colleagues found increased bone formation in trabecular and cortical bone in Ocn^−/−^ mice by bone histomorphometric analysis [63]. While OCN inhibits osteoblast function to inhibit bone formation, it is also thought to inhibit bone resorption by suppressing osteoclast function and is believed to be a common inhibitor of bone formation and bone resorption, reducing the renewal of old bone.

#### 3.3.2. Alkaline Phosphatase (ALP)

ALP is a plasma membrane-bound glycoprotein that is produced early in osteoblast mineralization and positively correlates with the rate of bone formation. Although the expression of osteogenic genes such as osteocalcin is upregulated late in mineralization, the expression of ALP decreases. BMP, Runx2, and Wnt cascade signaling were found to be the main pathways regulating ALP expression. ALP is required for bone matrix mineralization. During mineralization, osteoblasts first transport hydroxyapatite crystals to the matrix by secreting vesicles. Hydroxyapatite is then released from the vesicles to fill the pores between collagen fibers. During this stage, the ratio of Ca/P is important for the continued expansion of hydroxyapatite. ALP plays a role in the hydrolysis of inorganic pyrophosphate to produce inorganic phosphate. ALP deficiency causes hypophosphatasia, which leads to diseases of abnormal bone mineralization, such as rickets and osteochondrosis [64].

### 3.4. Bone Resorption Markers

#### 3.4.1. Anti-Tartrate Acid Phosphatase (TRAP)

TRAP is a glycosylphosphatidylinositol-anchored exoenzyme that belongs to the acid phosphatase family and is a characteristic marker of osteoclasts [65]. This enzyme is expressed on the folded membrane of osteoclasts, while its catalytic site is located within the acidic lumen formed between osteoclasts and bone. TRAP mediates the hydrolysis of luminal ATP and the reduction of luminal pH in the bone matrix and is involved in the resorption of the bone matrix by osteoclasts. As osteoclasts multiply and become more active, its expression increases.

#### 3.4.2. Cathepsin K (CTSK)

Cathepsin K is one of the most potent lysosomal cysteine proteases, and it is primarily responsible for promoting bone resorption [66]. Cathepsin K is mainly expressed in osteoclasts and other multinucleated cells. The helix and telopeptide regions of type I collagen can be cleaved by this enzyme. Cathepsin K is primarily regulated by the RANKL–RANK signaling pathway and is regarded as a marker of bone dissolving by osteoclasts. During bone resorption, osteoclasts secrete cathepsin K into the acidic bone resorption lumen formed by osteoclasts and the bone matrix. Once there, it is responsible for the degradation of type I collagen. Cathepsin K has been found to be closely associated with osteoporosis and is currently one of the most attractive targets for osteoporosis therapy.

## 4. Regulation of Bone Homeostasis by the Macrophage–Osteoclast Axis

### 4.1. Macrophages Are the Origin of Osteoclasts

**Figure 6:**
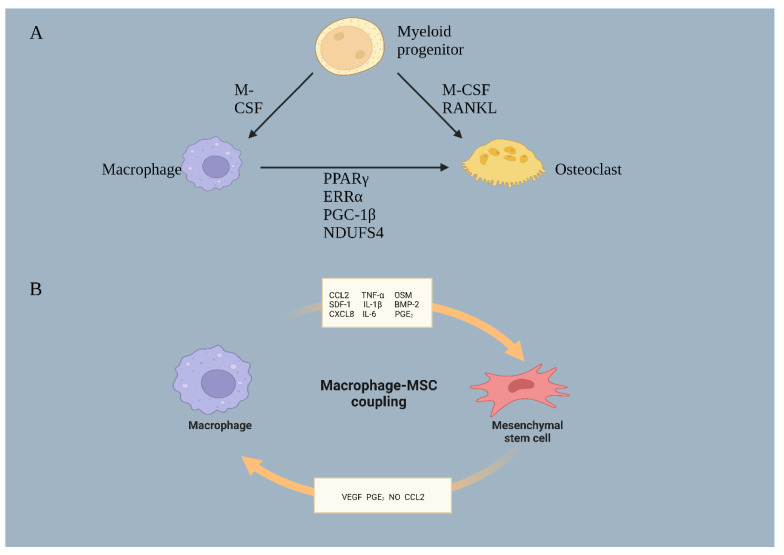
(**A**) Macrophage and osteoclast formation. Several regulatory factors such as PPARγ, ERRα, PGC-1β, and NDUFS4 mediate the differentiation of macrophages toward osteoclasts. (**B**) Interaction of macrophages with MSCs. Macrophages secrete a variety of cytokines to stimulate or inhibit the osteogenic differentiation of MSCs. In turn, MSCs can secrete cytokines that counteract macrophages to affect macrophage polarization and gene expression. By BioRender (SF24OOGRKM).

There is evidence that macrophages may also convert into osteoclasts capable of resorbing bone because osteoclasts come from the same ancestor as macrophages (Figure 6A). Normally, monocytes circulate in the peripheral blood and differentiate into macrophages once they are subjected to chemotactic factors to enter specific tissues. There is a robust regulatory function performed by macrophages in the immune response. Macrophages perform different roles in different tissues, which is known as macrophage diversity [67].

In the 1970s, Professor Walker’s pioneering experiments were the first to demonstrate that osteoclasts originated from hematopoietic cells [68]. Walker’s studies showed that the presence of precursor cells in hematopoietic cells that could differentiate into osteoclasts was essential for bone resorption. In 1990, Udagawa and his colleagues first demonstrated that mononuclear macrophages were the main source of osteoclasts [69]. Their results showed that after stimulation with M-CSF, bone marrow hematopoietic stem cells developed into monocyte/macrophage cells [70]. This can be followed by continued differentiation to form mature osteoclasts. Since then, osteoclasts have been classically regarded as the resident macrophage population in bone tissue.

### 4.2. Macrophage Fusion to Form Osteoclasts

It is essential to the formation of osteoclasts and the maintenance of bone mass that monocytes/macrophages fuse and multinucleate [71]. Monocyte/macrophage fusion leading to multinucleation to form osteoclasts is a complex, multistep regulatory process. Fusion during multinucleation includes several sequential steps, such as chemotaxis, membrane fusion, and reprogramming [72].

The chemotaxis and migration of macrophages are essential steps before cell membrane fusion. Several decades ago, scientists identified that osteoclasts are attracted by the chemoattractant 1 phospho-synuclein receptor (S1PR) [73]. Recent experiments have also pointed out the importance of chemokine ligand 2 (CCL2) and its receptor (CCR2) for the chemotaxis of osteoclasts as well [74]. Tight intermacrophage adhesion is also important for macrophage fusion. This adhesion is mainly related to integrin αvβ3. A deficiency of integrin αvβ3 impairs cytoskeletal organization and osteoclast resorption [75]. Integrins and integrin-related proteins are present in conical, actin-rich structures outside the macrophage membrane, called podosomes [76]. In contrast, in osteoclasts, podosomes form an actin ring (also called the confinement zone) that maintains the accumulation of metalloproteinases and the local acidic environment between the osteoclast and the bone surface [77].

Many genes have been acknowledged as participants in the membrane fusion proceeding of macrophages, comprising the dendritically expressed seven transmembrane proteins (DC-STAMP), CD44, CD47, and the tetraspanins CD9 and CD81 [70]. Notably, cell fusion between osteoclasts and macrophages is regulated by DC-STAMP, which is essential for cell fusion between osteoclasts and macrophages [78]. Moreover, it can be used to identify macrophage fusions. However, the ligand of DC-STAMP and its role within non-fused macrophages are still unclear.

The fused multinucleated cells are reprogrammed to eventually become mature osteoclasts. The hallmark of mature osteoclast formation is the expression of cathepsin K and MMP [79]. A significant role in bone remodeling is played by these two enzymes in breaking down the bone matrix. Furthermore, osteoclasts possess an enzyme known as anti-tartrate acid phosphatase (TRAP), which is widely used to determine differentiation. The staining intensity of TRAP increases when osteoclasts are multinucleated.

### 4.3. Regulators of Macrophage to Osteoclast Differentiation

#### 4.3.1. PPARγ

PPARγ plays an important role in the pro-inflammatory response and macrophage activation. The PPARγ protein is expressed in myeloid cells such as macrophages, and its activation is necessary for the maturation of macrophages with anti-inflammatory activity [80]. It was found that the deletion of PPARγ in the osteoclast lineage disrupts RANK–RANKL signaling, leading to osteosclerosis due to reduced osteoclast numbers and bone resorption [81]. In contrast, activation of PPARγ promotes bone resorption and osteoclast differentiation. In addition to its role in osteoclasts, PPARγ is also known to inhibit osteoblast differentiation and bone formation [82]. Osteoblasts exposed to oxidative stress increased the expression of PPARγ while decreasing the expression of RUNX2 and osteocalcin [83]. Thiazolidinedione (TZD), the most popular group of drugs for type II diabetes, has also been shown to induce PPARγ while causing patients to have weaker bones and significantly lower bone mineral density (BMD) [84]. Overall, PPARγ and its ligands promote osteoclast differentiation and bone resorption but inhibit osteoblast differentiation and bone formation, resulting in bone homeostasis.

#### 4.3.2. ERRα

It has also been shown that ERRα is a key mediator of macrophage and osteoclast functions in recent studies [85]. ERRα expression is upregulated in pro-inflammatory macrophages (M1) such as those induced by IFNγ or LPS, but its role is to suppress inflammation. It blocks the Toll-like receptor (TLR)-induced inflammatory response by transducing TNFα-inducible protein 3 (Tnfaip3) and controlling macrophage metabolic reprogramming [86]. ERRα has been demonstrated to play a role in osteoclast adhesion and migration. ERRα knockout mice phenotypically exhibit osteosclerosis due to osteoclast defects and reduced bone resorption and thus inhibits osteoclast maturation [85,87]. Statins or bisphosphonates can reduce cholesterol synthesis to decrease ERR and transcriptional activity while participating in the regulation of bone resorption and bone remodeling [83]. ERRα acts as a regulator of the balance between osteoclasts and macrophages, as well as a regulator of bone homeostasis.

#### 4.3.3. PGC-1β

Recent research indicates that PGC-1 may regulate macrophage and osteoclast function in a manner similar to PPAR and ERRα [88]. Activation of M2 macrophages is accompanied by induction of oxidative metabolism and expression of PGC-1β [89]. The effect of PGC-1β on M2 macrophage activation may be related to the promotion of mitochondrial biosynthesis and down-regulation of pro-inflammatory factors, as inhibition of oxidative mitochondrial respiration prevents macrophage polarization to the M2 phenotype [90]. It promotes osteoclastic differentiation as well as inhibits the generation of proinflammatory macrophages. In vivo and in vitro experiments demonstrated that downregulation of PGC-1β inhibited osteoclast mitochondrial biosynthesis and differentiation, and that osteoclast function was severely impaired in PGC-1β-deficient mice. Proper activation of PGC-1β is necessary for macrophage polarization and osteoclast differentiation.

#### 4.3.4. NDUFS4

NDUFS4 is located in the inner mitochondrial membrane and can inhibit the upregulation of macrophage pro-inflammatory genes [91]. However, it has the opposite effect on osteoclastogenesis in macrophages. We generally believe that inflammation is associated with osteoclastogenesis, but the inflammatory factor NDUFS4 is required for osteoclastogenesis, and its expression promotes osteoclast formation. Treatments for diseases associated with inflammatory bone injury may be effective by targeting NDUFS4 for its role in macrophage/osteoclast function.

### 4.4. Macrophages Support the Function of Osteoclasts

Macrophages may be involved in supporting osteoclast-mediated bone resorption in addition to fusing and differentiating into osteoclasts. Using a postmenopausal mouse model of osteoporosis, some researchers have found that macrophages remove bone resorption byproducts, including bone particles and TRAP, which are important for sustained bone resorption by osteoclasts [92,93]. We may be able to use this target to reduce bone loss in patients with osteoporosis. Regrettably, researchers did not explore whether macrophages can also support bone resorption by osteoclasts like this one in a normal bone homeostatic environment.

### 4.5. M1 Polarization and Activation of Osteoclasts

External stimuli and the disease environment have a profound effect on the activation of macrophages and osteoclasts. It is common for macrophages to activate into M1 cells under pathological conditions, releasing pro-inflammatory cytokines to fight disease-causing organisms. Especially after prosthetic implantation, the inflammatory microenvironment caused by the wear particles of the implant causes significant infiltration of M1 polarized macrophages, causing significant bone loss [94]. An important component of the inflammatory response of polarized M1 cells is the production of ROS, nitric oxide (NO), and the pro-inflammatory factors tumor necrosis factor-α (TNF-α), interleukin-6 (IL-6), and interleukin-1 (IL-1). Through these inflammatory factors, M1 polarized macrophages can directly or indirectly mediate the RANKL/RANK system to promote bone destruction by osteoclasts.

TNF-α is a famous pro-inflammatory factor that activates macrophages to release other inflammatory cytokines, such as IL-6 and IL-1β, to promote the inflammatory response. TNF-α promotes bone resorption in the regulation of bone homeostasis by increasing the expression of RANKL and M-CSF in osteoblasts, osteocytes, and stromal cells, as well as the sensitivity of osteoclast precursors to RANKL [95]. This mechanism of promoting RANKL-induced osteoclast formation may be achieved by inducing the expression of Blimp1. Different types of cells, including osteoclasts, need this transcriptional repressor to differentiate and function [96]. However, in the presence of M-CSF, TNF-α has been reported to stimulate osteoclast differentiation in the absence of RANKL and osteoblasts/myeloid cells [97]. This predicts that TNF-α has other effects on osteoclasts independent of the RANKL/RANK system. In addition to its great effect on osteoclast activation, TNF-α is one of the most potent inhibitors of osteogenic differentiation [98]. Recently, several studies have found that TNF highly expressed in M1 phenotype macrophages can upregulate E3 ubiquitin ligases. The C2-WW-HECT subfamily of E3 ubiquitin ligases includes Wwp1, Wwp2, Smurf1, Smurf2, Itch, and Nedd4. Wwp1 has been shown to mediate the degradation of Runx2 by binding to Shn3. Wwp1 mediates ubiquitination and degradation of JunB in response to TNF [99]. Runx2 and JunB are both key transcription factors regulating the osteogenic differentiation of MSCs. Smurf1, another protein of the C2-WW-HECT subfamily of E3 ubiquitin ligases, mediates ubiquitination and degradation of BMP signaling proteins and Runx2 and has a negative function on mature osteoblasts’ regulatory effect on the function of mature osteoblasts [100,101].

IL-1β is considered to be the most important family member of the IL-1 family, with solid pro-inflammatory activity by stimulating the synthesis of various pro-inflammatory mediators, such as cytokines, chemokines, and MMPs [102]. Macrophage production of IL-1β requires two signals: activation of Toll-like receptors to stimulate transcription and translation of the IL-1β gene, and induction of IL-1β release by NOD-like receptors [103]. Both IL-1α and IL-1β mediate bone resorption in vivo and in vitro by tingling the same receptor IL-1 receptor type I [104]. According to a recent study, the ability of TNF-α to stimulate RANKL production in osteoblasts is mediated by IL-1. In addition to stimulating the expression of RANKL in osteoblasts and stromal cells, IL-1 may also directly stimulate the differentiation of osteoclast precursors through a different pathway (RANKL-independent signaling). The RANKL-independent stimulation of osteoclasts may be induced by macrophage expression of the microphthalmia transcription factor (MITF) [105].

IL-6 is an important inflammatory cytokine that can be induced by IL-1β and TNF-α. In a model of disc degeneration, human disc cells treated with IL-1 had significantly higher IL-6 and IL-8 levels. IL-6 binds to its receptor (membrane-bound form or soluble form), which in turn promotes IL-1β expression [106]. The immune system responds rapidly to injury by producing IL-6 and releasing it into the bloodstream [107]. The interaction of osteoblasts on osteoclasts may be the main way in which IL-6 promotes osteoclast differentiation. It has been demonstrated that osteoblasts and osteoclasts efficiently stimulated by IL-6 can produce RANKL [108]. Through interactions between the IL-6/IL-6 receptor and glycoprotein 130 (gp130) receptor, Janus-activated kinase (JAK) is activated, and STAT3 is phosphorylated, promoting osteoclast formation. STAT3 is thought to control osteoclast differentiation and bone homeostasis through NFATc1 transcription [109]. Notably, there is evidence that the potential of IL-6 to stimulate osteoclastogenesis depends on the presence of IL-6 receptors on osteoblasts, as IL-6 does not significantly stimulate osteoclast formation unless osteoblasts are co-cultured with osteoclasts [110].

### 4.6. M2 Polarized and Osteoclasts

The M2 phenotype is primarily observed in macrophages under non-inflammatory conditions, where it promotes tissue repair and homeostasis. It is M2 macrophages that are responsible for clearing apoptotic cells and producing anti-inflammatory cytokines during the waning phase of inflammation. M2 macrophages produce anti-inflammatory factors, such as chemokine (CC motif) ligand 18 (CCL-18), CCL-22, IL-10, and, to a lesser extent, IL-12. Furthermore, M2 macrophages produce significant quantities of osteogenic growth factors, such as BMP-2, TGF-β, bone-bridging protein, and 1,25(OH)_2_ vitamin D_3_. It is well known that L-4 and IL-13 are anti-inflammatory cytokines responsible for polarizing macrophages toward the M2 state. In addition to reducing the expression of RANKL signaling, IL-4 directly prevents osteoclast formation by inhibiting the activation of NF-κB and mitogen-activated protein kinase through an independent mechanism [111]. Although IL-4 affects osteoclast formation, it may also hinder the differentiation of MSCs toward osteoblasts. A prominent example is the reduced production of osteoblasts in a model of MSCs that continuously secrete IL-4 [112].

IL-10, as an anti-inflammatory factor, is often associated with M2. After stimulation of macrophages by immune complexes via Fc receptors, M1 macrophages produce IL-10, but M2 produces more IL-10. IL-10 makes macrophages more sensitive to IL-4 and IL-13 by increasing cell surface IL-4Rα expression [113]. This makes macrophages more susceptible to differentiation into M2, which is regulated by STAT3 activated by IL-10R or IL-6R [114]. As an anti-inflammatory cytokine, IL-10 inhibits the synthesis of various inflammatory cytokines, including IL-1β, IL-6, and TNF-α [115]. IL-10 inhibits osteoclast activity by blocking the production of these pro-osteoclastic cytokines. Meanwhile, several studies have demonstrated that IL-10 directly prevents osteoclast formation in several assays [116]. In fact, by interfering with NFATc1 activation and nuclear translocation, IL-10 is a powerful inhibitor of osteoclastogenesis [117].

BMP-2 belongs to the family of BMP proteins. As opposed to M0 or M1-type macrophages, M2 is the major secretor of BMP-2. BMP-2 regulates RANKL and M-CSF synthesis, which is crucial for osteoclast survival, maturation, and differentiation. BMP-2 also activates the NFATc1 transcription factor, a key transcription factor for osteoclast differentiation [118]. As well as stimulating bone mineralization, BMP-2 has been shown to stimulate the differentiation of MSCs into mature osteoblasts [119]. Logically, Smad1 is phosphorylated and activated by BMP-2 binding to the BMP receptor, leading to Runx2 translocation to the nucleus of pre-osteoblast cells to upregulate osteogenic factors, including ALP and OCN [120]. BMP-2 promotes both osteoclast activation and osteoblast formation, accelerating the replacement of old bone.

## 5. Regulation of Bone Homeostasis by the Macrophage–Mesenchymal Stem Cell Axis

### 5.1. Macrophages Affect the Activity of MSCs

In addition to osteoclasts, macrophages also interact with MSCs to mediate bone homeostasis (Figure 6B). As shown in their first study, Champagne and his colleagues were able to increase the activity of ALP in human MSCs using a conditioned medium from non-activated J774A.1 murine macrophage cells [121]. This pioneered the idea that macrophages affect the osteogenic activity of MSCs. Subsequently, they demonstrated that this promotion of the conversion of MSCs to osteoblasts was mediated by BMP-2. The researchers used LPS to induce an inflammatory phenotype, which reduced osteogenesis and BMP-2 expression in J774A.1 cell, but they did not examine M2 polarization’s role in osteogenesis. Subsequently, similar results were found by Pirraco et al. They used inactivated human monocytes co-cultured with human bone marrow-derived MSCs. In their study, monocytes increased MSCs proliferation, ALP activity, OCN production, and bone-bridging protein expression, all of which were partially mediated by BMP-2 [122]. Nicolaidou et al. also found that the co-culture of inactivated human peripheral blood monocytes (PBMCs) and differentiated macrophages with human bone marrow stem cells demonstrated dose-dependent increases in bone formation and that there was an increase in bone formation in cultures with a more significant number of macrophages [123]. They subsequently found that monocytes secrete Oncostatin M (OSM) in response to direct intercellular contact with MSCs, which contributes to this effect. Similar results were found in an in vitro 3D culture model, with the addition of macrophages, regardless of their polarization status, increasing the osteogenic differentiation of MSCs compared to MSCs alone [124].

### 5.2. M1 Macrophages Directly Affect MSCs

M1 is well known for secreting inflammatory cytokines that inhibit the differentiation of MSCs into osteoblasts. However, the role of M1 in bone homeostasis is not exclusively to inhibit the differentiation of MSCs, but it has also been shown to promote the osteogenic differentiation of MSCs. Omar et al. found that stimulation of human monocytes with LPS or other Toll-like receptor activators enhanced bone formation in human MSCs [125]. Studies on the mechanisms involved suggest that Toll-like receptor activation induces monocyte production of Oncostatin M (OSM) through induction of COX-2 and prostaglandin E2 (PGE_2_) and is responsible for bone formation through OSM signaling of gp130 on MSCs. This may partly contribute to the potential adverse effects of NSAIDs on fracture healing. Subsequently, a co-culture system was used by Lu et al. to investigate whether M0, M1, and M2 macrophages can induce osteogenic differentiation in mouse bone marrow MSCs [126]. While all other macrophage subtypes exhibited some bone formation enhancement, a strong effect was observed for the M1 macrophage subtype. Three-dimensional culture models also showed that pro-inflammatory M1 macrophages most effectively enhanced new bone formation [122]. In line with the results of this experiment, Guihard and colleagues observed that it was mainly M1 macrophages that produced OSM [127]. Interestingly, the effect was dose-dependent, with the best osteogenesis-promoting effect being a macrophage-to-MSCs ratio of 5:1. However, in the macrophage–MSC co-culture system, there is a negative relationship that exists between OPG and M1 macrophages, which suggests that further studies are needed to determine the significance of the OPG–RANKL ratio and its relationship to M1 macrophages’ function in osteoclastogenesis. The stimulatory effect of pro-inflammatory macrophages on MSC osteogenesis was explained from another perspective by Tu et al. [128]. IL-23 secreted by macrophages directly induces osteogenesis in MSCs through the activation of STAT3 and β-linked proteins. In response to the neutralization of IL-23 in macrophage CM by IL-23 p19 antibody, both calcium formation and ALP activity are reduced in MSCs. It has also been found that the cytokines CCL2 and VEGF secreted by M1 promote the recruitment of BMSCs, which is required for the subsequent repair of the bone injury site [129].

The shift between M1 and M2 in bone homeostasis and bone healing has attracted a lot of attention. In previous studies, it has been shown that injuries induce immune responses that are necessary for repair progression during the pro-inflammatory phase. Bone healing is severely impaired in animals with macrophage depletion during the pro-inflammatory phase [130]. Treatment with 1,25(OH)_2_ vitamin D_3_ during the inflammatory phase inhibited M1 macrophage function while promoting M2 macrophages but hindered fracture repair. It is believed that macrophages were primarily involved in the inflammatory response at the site of injury, but the number of M1 macrophages decreased gradually after day seven of the injury. As the M1 to M2 ratio decreases after day seven, it indicates that the M1 to M2 transitions are critical for improved osseointegration. The results of Nathan et al. showed that a proinflammatory environment of 72 to 96 h is essential for proper MSC osteogenesis [131].

M1-polarized macrophages play different roles in bone homeostasis. They induce apoptosis in MSCs, but also promote osteogenic differentiation in MSCs via COX-2-PGE_2_ signaling. Whether M1-polarized macrophages exert an osteogenesis-inhibiting or bone-repair-promoting role may depend mainly on the number of different cytokines it secretes. Previous designs of bone regeneration studies have focused on enhancing the polarization of M2, but neglected the significance of the early M1 presence. We should clearly recognize the necessity of M1-polarized macrophages for osteoclast activation and initial induction of MSCs. As a whole, normal bone homeostasis is regulated by the M1 macrophages, which are essential and necessary for the initiation of the proinflammatory phase.

### 5.3. M2 Macrophages Directly Affect MSCs

Regulation of the inflammatory phase is fundamental for common bone repair; in contrast, persistent inflammation is detrimental to fracture healing outcomes. As is known to us all, M2-polarized macrophages promote tissue repair and bone formation. Gong et al. studied a co-culture system of mouse macrophages and MSCs in which M2 significantly enhanced the osteogenic differentiation of MSCs, whereas M1 impaired it [132]. Upregulation of the growth factors TGF-β, VEGF, and IGF-1 was observed in M2 macrophages, whereas upregulation of deleterious inflammatory cytokines, including IL-6, IL-12, and TNF- α, was observed in M1 macrophages. In a study of bone defect healing, researchers found that M2 macrophage polarization enhanced bone formation and osteogenesis by MSCs in an IL-10-dependent manner [133]. Zhang et al. confirmed that increased OSM production in M1 cultures stimulated early and mid-stage osteogenic differentiation of MSCs without enhancing matrix mineralization. On the contrary, increased BMP-2 production in M2 macrophage co-cultures in direct and indirect co-culture systems was more favorable for the mineralization of MSCs, i.e., the late stages of osteogenesis [134]. Specifically, these studies suggest that inflammatory cells of the M1 subset participate in the initial acute phase of a fracture and remove debris from the fracture site, stimulating only osteogenic differentiation of MSCs in the early and middle stages of co-culture. The M2 macrophage supports MSC-intervened bone formation rehabilitation in the late fracture phase, promoting the proliferation and osteogenic differentiation of MSCs.

### 5.4. Macrophages Affect MSCs through Exosomes

Exosomes are tiny 30–120 nm extracellular vesicles originating from the plasma membrane that serves as an essential mediator of intercellular communication, carrying molecular cargo and transferring bioactive components. Activation signals originating from M2 macrophage exosomes can promote miR-690 levels in BMSCs and upregulate osteogenic differentiation [123]. Arjen et al. also reported that MC-EVs upregulates the expression of genes encoding matrix metalloproteinases (MMPs) and increase the secretion of CXC, thereby stimulating processes associated with ECM structural reorganization and thus promoting osteogenic differentiation [135]. Interestingly, because macrophage-derived exosomes are tightly regulated, the inflammatory environment may not alter its osteogenic effects. Interleukins associated with the inhibition of osteogenesis were not detected in exosomes released from LPS-stimulated macrophages in a culture medium. It has also been demonstrated that exosomes produced by LPS-activated macrophages improve the secretion of Runx2 and BMP-2 in BMSCs and promote osteogenic differentiation [119]. This suggests that exosomes released from macrophages may have a role in regulating bone homeostasis that is not identical to that of macrophages. This may be related to the selective assembly of exosomal components.

### 5.5. MSCs Directly Affect Macrophages

In addition to regulating the function of macrophages, MSCs can also influence macrophage polarization, phagocytosis, and metabolism. Studies have shown that MSCs may induce macrophages to convert to an anti-inflammatory/immunosuppressive phenotype. MSCs produce an anti-inflammatory response due to inflammatory factors secreted by macrophages. MSCs were found to exhibit immunomodulatory activity by suppressing M1 and enhancing M2 macrophage phenotypes in a three-dimensional in vitro bone model [122]. In the co-culture system, MSCs significantly inhibited the production of pro-inflammatory factors secreted by M1 and promoted IL-10 secretion by macrophages [136]. Mechanistic studies have shown that the immunosuppressive properties of MSCs are triggered by interferon γ (IFNγ) and other pro-inflammatory cytokines such as TNFα, IL-1α, or IL-1β [137]. MSCs recognize these inflammatory factors and then mediate immune regulation through iNOS and COX2-dependent pathways to enhance the production of PGE_2_. PGE_2_ is a major product of arachidonic acid metabolism in mammalian cells, and it is essential for immunosuppression and the suppression of inflammation [138]. PGE_2_ production alters macrophage metabolic status and increases the secretion of IL-10 through its binding to EP2 and EP4 receptors [139]. Using MSCs co-cultured with macrophages, Cho et al. found that M1 polarization was significantly prevented, and M2 polarization was significantly induced [140]. MSC-secreted TGF-β was shown to decrease the levels of M1 markers, upregulate the levels of M2 macrophage markers, and inhibit the over-activation of inflammatory responses in the co-culture system.

As well as direct immunomodulatory effects on macrophages, MSCs also regulate macrophage chemotaxis, which is necessary for MSC-mediated immunomodulation. MSCs secrete a large number of several chemokines, including CCL2 and CCL4 [141]. MSCs affected by inflammatory cytokines significantly increase the secretion of chemokines such as CCL2, which induce macrophage recruitment and repair of tissues.

### 5.6. MSCs Affect Macrophages through Exosomes

The regulation of macrophages by MSCs was shown to be carried out to a large extent through exosomes. Compared to other cells, the exosome output of MSCs is enormous and, therefore, the most extensively studied. MSCs-derived exosomes can be powerful tools for macrophage recruitment and polarization. Furuta and his colleagues experimentally found that MSCs-derived exosomes enhance osteogenic differentiation and angiogenesis [142]. Xu et al. also found that BMSC-derived exosomes have an important role in lipid polysaccharide (LPS)-stimulated in vitro conversion of macrophage subpopulations from M1 to M2 phenotype [143]. Shi et al. found that local administration of MSCs-derived exosomes reduced infiltration of M1 macrophages and expression of the pro-inflammatory factors IL-1β and IL-6 during the acute inflammatory response of tendon–bone healing [144]. In addition, miR-21 in MSCs-derived exosomes was found to promote osteogenic differentiation and improve fracture healing [139]. Mitochondrial transfer studies have also shown that MSCs-derived exosomes can transfer mitochondria to macrophages to promote phagocytosis and inhibit the secretion of inflammatory factors [145]. Overall, MSCs-derived exosomes, like MSCs in general, regulate M1 to M2 conversion, maintain the presence of M2, and promote tissue repair and bone formation.

Targeting the ability of MSC exosomes to regulate M1 and M2 polarized macrophages, researchers can also pre-treat MSCs for more powerful functions. For example, in a study of wounds in diabetic patients, researchers used melatonin to pre-stimulate MSCs and confirmed that their secreted exosomes could regulate the polarization of M1/M2 macrophages through the PTEN/AKT pathway to obtain excellent wound healing [146]. In addition, MSCs-derived exosomes after hypoxia preconditioning and inflammatory environment preconditioning were also shown to regulate M1/M2 polarization and promote tissue damage repair through different pathways [147,148,149]. We can also borrow this idea and use MSCs-derived exosomes after drug stimulation to regulate bone homeostasis.

## 6. Effect of the Macrophage–Osteoblast Axis on Bone Homeostasis

Macrophages in bone are on the surface of the bone, in close proximity to mature osteoblasts at the site of bone remodeling, which predicts that macrophages may have a direct impact on osteoblast function [150]. Mice with defective macrophages had reduced numbers of mesenchymal progenitor cells, and the ability of these mesenchymal progenitor cells to differentiate into osteoblasts was decreased further [151]. Macrophages have been shown to be required for osteoblast mineralization of the bone matrix, and macrophage-dependent osteoblast differentiation has been shown to be dependent on BMP-2 and TGF-β signaling [152,153]. Macrophages also promote the synthetic effects of parathyroid hormone (PTH), a key regulatory hormone of bone homeostasis, in bone tissue [154,155]. Macrophages in white adipose tissue are involved in the secretion of osteopontin, which may be a potential pathway for the treatment of obesity-associated bone disease [156]. Thus, macrophages may further coordinate bone homeostasis through the macrophage-osteoblast axis.

### 6.1. M1 Macrophages Affect Osteoblasts

Regulation of osteoblasts by macrophages depends mainly on the secretion of specific cytokines. As mentioned above, M1-polarized macrophages are a major source of inflammatory cytokines, such as TNF-α. Chronically elevated TNF-α has been shown to be closely associated with a variety of inflammation-related diseases, such as osteoporosis [157]. TNF-α possesses two receptors, including TNFR1 and TNFR2 [158]. TNFR1 is expressed in most tissues, and its activation leads to apoptosis. TNFR2 is mainly expressed in immune-related cells, and its activation leads to cell proliferation. In osteoblasts, several research groups have reported the inhibitory effect of TNF-α on osteogenic differentiation [159,160,161,162]. Studies on its mechanism have found that inflammatory cytokines inhibit the differentiation of osteoblasts mainly by suppressing the expression of osteogenic factors (Runx2 and IGF-1) in osteoblasts. However, other studies have shown a dual action of TNF-α on osteoblasts, which also stimulates the osteogenic potential of osteoblasts. These opposing experimental results may be due to the dose-dependent effect of TNF-α on osteoblasts. It has been shown that low concentrations (0.5 ng/mL) of TNF-α significantly promoted osteoblastic differentiation of osteoblasts in vitro [163]. It increased the expression of osteoblast Runx2 and BSP activity, and the expression of TNFR2, the receptor of TNF-α, was elevated. In contrast, high concentrations of TNF-α are negative regulators of osteogenic differentiation. In conclusion, low and high concentrations of TNF-α have completely different effects on osteoblasts, and further studies are needed. Regulation of inflammatory factors may be a potential option for the treatment of disorders of bone homeostasis, such as osteoporosis.

### 6.2. M2 Macrophages Affect Osteoblasts

As an anti-inflammatory cell, M2 not only secretes anti-inflammatory cytokines but also secretes a certain amount of osteogenic inducer BMP-2. BMP-2 belongs to the BMP protein family, which can effectively stimulate osteoblast differentiation and bone mineralization. The binding of BMP-2 to the BMP receptor induces phosphorylation of Smad1, leading to Runx2 translocation into the nucleus to upregulate the expression of osteogenic factors, such as ALP and OCN. BMP-2 is secreted much more strongly by macrophage M2 than macrophages M0 and M1, contributing to the induction of osteogenic differentiation in M2. There is evidence that osteoporosis may be caused by BMP-2 deficiency [164,165,166]. This may be because the rate of bone remodeling decreases when BMP-2 is reduced, and old bone is not replaced as well. Further studies revealed that, in addition to BMP-2, TGF-β is also a potential key factor in promoting osteoblast formation during bone homeostasis [129]. On the other hand, Batoon et al. also found that CD169^+^ macrophages are essential for osteoblast maintenance and bone homeostasis and promote intramembranous and endochondral ossification during bone repair [167]. In conclusion, M2-polarized macrophages have an important role in promoting bone remodeling and maintaining bone homeostasis. A timely, well-regulated, and coordinated transition of the immune microenvironment from pro-inflammatory to anti-inflammatory in the region of the fracture is necessary to promote the regeneration of the injured bone.

## 7. Regulation of Bone Homeostasis by the Macrophage–Osteocyte Axis

Osteocytes have a crucial role in normal bone homeostasis and may regulate the differentiation of osteoclasts and osteoblasts. Osteocytes can promote RANKL production and increase the RANKL/OPG ratio, causing enhanced osteoclastogenesis and bone resorption. In the macrophage–osteocyte axis, macrophages can mediate bone homeostasis by regulating osteocyte activity. Oxidative stress caused by aging and inflammation leads to M1 polarization of macrophages and the production of inflammatory factors. IL-1 is known to promote loss of osteocyte viability through NF-kB/RANKL signaling. As one of the basic components of bone, massive apoptosis of osteoblasts causes massive loss of bone mass. In a mouse model of estrogen deficiency, osteocyte autophagy is increased under oxidative stress conditions [168]. This is also induced by the M1 polarization of macrophages. Briefly, overexpression of macrophage inflammatory factors induces abnormal apoptosis of osteocytes, leading to osteoporosis or bone loss.

Macrophages also mediate the chemotaxis of osteoclasts after osteocyte death. Andreev et al. found that macrophage-induced C-type agglutinin (Mincle) could be activated by DAMP released from dying osteocytes [169]. Mincle enhances osteoclastogenesis through ITAM-based calcium signaling and oxidative phosphorylation induction. Inhibition of Mincle may be an effective way to uncouple osteoclast activation and osteocyte death.

## 8. Regulation of Bone Homeostasis by the Macrophage–Blood/Vasculature System Axis

In addition to interacting with components in BMUs, macrophages also affect bone homeostasis through the blood/vasculature system. Macrophages secrete VEGF and HIF in large amounts to promote the growth of H-type vessels, which has been shown to play a vital role in bone homeostasis [170,171]. H-type vascular endothelial cells mediate the local growth of the vascular system and provide ectopic signals to perivascular osteoprogenitor cells. In contrast, the decline of H-vessels and the concomitant decrease in osteoprogenitor cells during aging may provide a plausible explanation for the decrease in bone mass. Another team of researchers explained the effect of macrophages on H-type blood vessels from another direction [172]. They found that TRAP^+^ macrophages induce transcriptional expression of periostin and recruitment of periosteal-derived cells (PDCs) to the periosteal surface through the secretion of PDGF-BB. These recruited PDCs undergo osteogenic differentiation to promote cortical bone formation and are accompanied by H-type angiogenesis.

In addition to the vascular system, macrophages also interact with red blood cells to regulate bone homeostasis. Studies have shown a functional link between erythropoiesis and bone homeostasis. Fractures are more likely to occur and bone mineral density is lower in patients with anemia [173]. Although the specific mechanisms involved are currently unknown and may be related to the remodeling of the bone marrow microenvironment, macrophages can regulate bone homeostasis by participating in the erythropoietic process. In addition to the macrophages adjacent to the osteoblasts in bone, there are central macrophages which, together with the surrounding erythrocytes, form the basis of the erythroblastic islands (EBIs). An erythroblastic island in the human body is a structure that facilitates the differentiation of myeloid lineage progenitors into reticulocytes and has been found at multiple sites of erythropoiesis in humans [174]. Central macrophages promote the development of erythroid cells in erythroid islets by secreting stimulatory cytokines, such as IGF-1 and BMP-4 [175]. Macrophages are also involved in the clearance of mature erythrocyte nuclei. Specifically, immature central macrophages secrete growth factors that promote the proliferation of erythroid precursor cells and their differentiation. In contrast, mature central macrophages provide iron and phagocytose nuclei in order to promote the maturation of late-phase erythroid cells. From a different perspective, macrophages offer large amounts of Fe^2+^ to erythrocytes via ferroportin (Fpn). Fe^2+^ is associated with the regulation of osteoclast function and Fe^2+^ release via Fpn is necessary for normal osteoclastogenesis and overall skeletal homeostasis in mice [176]. For example, mice lacking Fpn activity in osteoclasts accelerated osteoclastogenesis and bone resorption [177]. These findings suggest that macrophages have the potential to coordinate erythropoiesis and bone homeostasis.

## 9. Possibility of Treating Disorders of Bone Metabolism and Bone Defects by Macrophages

Disorders of bone homeostasis, represented by osteoporosis and aging, are characterized by excessive activation of bone resorption and diminished bone formation. The search for mechanisms regulating bone homeostasis has been ongoing for many years with the aim of finding better treatments for disorders of bone homeostasis. In the past, there has been a focus on the direct link between osteogenic spectrum cells and osteoclasts. As research proceeds, the results of an increasing number of in vitro and in vivo studies illustrate the necessary interactions between bone regeneration and polarized macrophages during the bone healing process. Macrophages, as an essential factor in the regulation of bone homeostasis, may be a relevant target for therapy. The role of macrophages in bone homeostasis is complex, and it is clear that their role ranges from subtle and precise subtypes to switching between different subtypes. Overall, immunomodulatory approaches are potential tools to promote endogenous regenerative capacity in high-risk patients. For this type of approach, macrophages represent a promising cellular target. Promoting bone regeneration over bone resorption through macrophage strategies may also be a powerful potential approach to promote bone defect repair. Researchers have developed methods to load macrophage regulatory factors onto biomaterials for delivery into the body to regulate bone homeostasis. Wang et al. used PTH-related peptides to repair bone defects as well as achieve good bone regeneration by regulating the polarization of macrophages [178]. Chen et al. alleviated periodontitis using the HIF-1α activator DMOG to inhibit bone resorption by regulating macrophage polarization [179]. Luo et al. sequentially regulated the polarization of macrophages through bone tissue engineering materials to promote the repair of bone defects to a greater extent [180]. Liu et al. also utilized macrophage-derived extracellular vesicles to recruit more BMSCs and significantly promote the repair of bone defects [181]. This provides new insights into the use of macrophages to regulate bone homeostasis. However, due to their functional complexity and plasticity, there is a need to decipher the underlying cellular and biological mechanisms of macrophage function during bone homeostasis.

## 10. Summary and Future Perspectives

Bone homeostasis is a complicated and sophisticated process in which multiple regulatory mechanisms work in concert. Macrophages regulate bone homeostasis through multiple molecular mechanisms via the macrophage–osteoclast axis, macrophage–MSCs axis, macrophage–osteoblast axis, macrophage–osteocyte axis, and macrophage–blood/vasculature system axis. In addition to the differential expression of activated macrophages critical for normal bone homeostasis, macrophages play a key role in oxidative stress-driven skeletal diseases. Osteoporosis or bone loss is always associated with systemic inflammation and the M1 polarization status of macrophages. However, a mild inflammation state may also benefit bone formation. Polarized M2 macrophages can induce osteoblast differentiation and increase bone mineralization. Macrophage-based therapeutic strategies appear to be a possible key target for inducing bone regeneration and reducing bone loss. Modulating the cytokine profile in the local microenvironment to favor the M2 polarization of macrophages could be a novel strategy to improve bone loss. Blocking macrophage-induced osteoclastogenesis and promoting macrophage-induced osteoblast production also contribute to bone regeneration. Biomaterial-based macrophage modulation may also provide a potential treatment for fractures and bone defects. Loading macrophage regulatory factors into bone repair scaffolds to regulate macrophage polarization and related factor expression will contribute to rapid repair of fractures or bone defects. In conclusion, normal bone homeostasis is the result of inter-crosstalk between macrophages and multiple cellular components. The strategy of macrophages to regulate bone metabolism or promote bone defect repair may be an important issue for future development.

## Figures and Tables

**Table 1:** Origin, function, and signaling of multiple cells in bone.

Cell	Origination	Function	Activation Signals	Transcri-Ption Factors	References
Osteoclast	Monocyte/macrophage	Resorption of broken old bone and control of bone mass	Macrophage colony-stimulating factor (M-CSF), receptor activator of nuclear factor κB ligand (RANKL)	NFATc1	[18,19,20,21,22,23,24]
BMSCs	-	Multidirectional differentiation potential, regulation of osteoclast differentiation and macrophage polarization	-	-	[25,26]
Osteoblasts	BMSCs	Bone matrix formation and mineralization, secretion of multiple bone matrix proteins, initiation of osteoclast bone resorption	Bone morphogenetic protein (BMP), insulin-like growth factor (IGF), transforming growth factor (TGF), and the Wnt pathway	Runx2, Osterix (OSX)	[15,27,28,29,30]
Osteoocytes	Osteoblasts	Sensing mechanical forces, regulating bone mass, and producing chemotactic signals from osteoclasts, release of SOST to prevent bone overproduction	-	-	[27,31,32,33,34,35,36]
Macrophages	Monocyte lineage	Phagocytosis of pathogens, presentation of antigens, secretion of cytokines, induction of immune responses, and regulation of tissue homeostasis	Lipopolysaccharide (LPS)	nuclear factor kappaB (NFκB)	[37,38,39]

## Data Availability

Not applicable.
